# Effects of Dynamic Perturbation-Based Training on Balance Control of Community-Dwelling Older Adults

**DOI:** 10.1038/s41598-018-35644-5

**Published:** 2018-11-22

**Authors:** Jo-En Chien, Wei-Li Hsu

**Affiliations:** 1 School and Graduate Institute of Physical Therapy, College of Medicine, National Taiwan University, Taipei, Taiwan; 2 Physical Therapy Center, National Taiwan University Hospital, Taipei, Taiwan

## Abstract

Walking is one of the daily activities that may cause falling in older adults. We developed a novel dynamic balance training program using a perturbation-based training on a custom-made treadmill, which can generate forward, backward, and lateral sway perturbations during walking. The purpose of this study was to investigate the changes in the balance performance of community-dwelling older adults after 8-weeks of perturbation-based balance training. A three-dimensional motion analysis system was used to collect kinematic and kinetic data. Seventeen community-dwelling older adults performed quiet standing with and without the balance perturbation. Biomechanical parameters such as center of pressure (COP) and center of mass (COM) were calculated. A paired t-test was used to compare the difference in balance performance before and after the training. After training, the results showed that the COM control of the older adults was significantly improved during quiet standing with perturbation, while the COP control during quiet standing without perturbation was not changed. The perturbation-based balance training exerted a positive effect on dynamic balance control in older adults. This translational research offers a new paradigm of balance training and can be applied to patient populations who have a high risk of falling.

## Introduction

Fall-induced injury is the major risk factor of injury-related deaths in adults aged 65 years or older^1^. Most falls occur during walking; therefore, fall prevention during walking has become an important topic for older adults^2^. However, in previous studies, balance training mostly focused on muscle strengthening or static balance exercises^3,4^. Recent studies have proven that dynamic balance training improves balance performance and decreases falls^5–9^.

The risk factors for falling can be generally classified into intrinsic factors and extrinsic factors. Intrinsic factors are related to patients, such as dizziness or lower extremity weakness while extrinsic factors are associated with the environment, such as slips, trips, or falling from stairs^10^. A previous study showed that almost 60% of community-dwelling adults experienced extrinsically related falls^11^. Thus, we developed a novel training program by using a perturbation-based training with a custom-made treadmill, which can generate forward, backward, and lateral sway perturbations during walking to mimic an external risk factor of falling.

## Effects of Ageing on Balance Control

Balance control can be divided into three parts: biomechanical structure, sensory organisation, and motor coordination^12^. Because of ageing, older adults might change their movement strategies to accommodate physiological deconditioning. Within the biomechanical structure, low muscle strength and low bone mineral density with kyphosis increase the risk of falling in older adults^13,14^. With regard to sensory organisation, older adults often have proprioception deficits in position and motion sense; therefore, they rely more on visual cues when walking^15,16^. In motor coordination, older adults often rely on hip and stepping strategies to adjust their steps to overcome unexpected disturbances^6,14,17,18^. All aforementioned reasons increase energy consumption and increase the risk of falling.

Visual, vestibular, and proprioception systems contribute to quiet standing, which is often used to assess static postural control^19–21^. The postural sway in quiet standing can be quantified using center of pressure (COP) parameters^22–28^. A previous study showed that the COP excursion, velocity, and distance were higher in older adults than in young adults^29^. Moreover, the center of mass (COM) has been used to estimate the whole-body balance control and the older adults often use a rigid pattern to control the movement of the COM^18,30,31^. Therefore, the COM and COP are reliable parameters for evaluating postural stability and upright balance control.

## Perturbation-based Balance Training

Previous studies have used various methods to provide balance perturbations to challenge body’s postural stability, such as waist pulling^32^, mastoid vibration^33^, a rotational treadmill^34^, decelerated walking speed^8^ and a movable platform^5,7,35,36^. The results have shown that the effect of perturbation-based training may decrease the incidence of falls^5^. However, most of these studies have only provided single-plane balance perturbation and could not quantify the amplitude of perturbation.

A perturbed walking exercise can mimic real slips during walking. Previous studies have shown that perturbation-based training using a split-belt treadmill which provided an anterior-posterior direction of balance perturbation can reduce the likelihood of falls during level-ground walking, improve dynamic balance control^37^, and decrease the reaction time to an auditory stimulus while walking^8^. However, falls often occur as a result of incorrect weight shifting and excessive trunk sway during walking^2^, and no study has included both anterior-posterior perturbation and lateral sway perturbation in a perturbation-based training on a treadmill.

Therefore, the aim of this study was to investigate the static and dynamic balance control of community-dwelling older adults after they received 8-week perturbation-based treadmill balance training that focused on both anterior-posterior and lateral sway perturbations. The research hypothesis was that the balance control of older adults improves after 8 weeks of perturbation-based treadmill balance training.

## Results

A three-dimensional motion analysis system was used to collect kinematic and kinetic data. Seventeen community-dwelling older adults performed quiet standing with and without the balance perturbation. Biomechanical parameters such as center of pressure (COP) and center of mass (COM) were calculated. A paired t-test was used to compare the changes in balance performance before and after the training.

There were a total of 17 participants in the study (12 women and 5 men; mean age 68.33 ± 5.80 years old; Skewness: −0.158, Kurtosis: −1.218, Shapiro-Wilk test: 0.468). The average height was 157.09 ± 5.35 cm (Skewness: 0.158, Kurtosis: −1.218, Shapiro-Wilk test: 0.468) and the average weight was 58.92 ± 7.69 kg (Skewness: 0.210, Kurtosis: 1.699, Shapiro-Wilk test: 0.178). The normality hypothesis of the participants’ demographic data was verified by means of the Shapiro-Wilk test and rejected at the significance level of 0.05. Two out of 17 participants had experienced a fall during the last 12 months. Their Mini-Mental State Examination score was 28.82 ± 1.19.

### Quiet Standing

After receiving 8 weeks of perturbation-based training, participants’ performance were varied during static and dynamic quiet stance. No serious falls were occurred during training or data collection for all participants.

#### Without balance perturbation

During quiet standing without perturbation (static condition), all COP parameters including COP excursion, range, displacement, velocity, and sway area were not significantly changed after the training (Table 1). Neither eyes opened nor eyes closed were different in all COP parameters.

**Table 1:** Center of pressure (COP) parameters during quiet standing without perturbation (static condition) before and after the training.

Variables		Eyes open							Eyes closed						
		Before		After		Statistic			Before		After		Statistic		
		Mean	SD	Mean	SD	t	p	*ES*	Mean	SD	Mean	SD	t	p	*ES*
Excursion (mm)	AP	3.81	1.59	4.00	2.60	−0.43	0.67	−0.09	4.06	1.87	4.34	2.59	0.47	0.64	−0.12
	ML	1.64	1.28	1.68	1.81	−0.17	0.87	−0.03	1.34	0.67	1.42	1.37	−0.23	0.82	−0.07
	RD	4.48	2.08	4.77	3.05	−0.64	0.53	−0.11	4.53	1.93	4.90	2.79	−0.61	0.55	−0.15
Mean Velocity (mm/sec)	AP	6.67	1.71	6.39	1.95	0.69	0.50	0.14	8.54	2.37	9.34	3.23	−1.14	0.27	0.28
	ML	3.57	1.76	3.38	1.90	0.94	0.36	0.10	3.34	1.12	3.38	1.27	−0.11	0.91	0.03
	RD	8.31	2.37	7.92	2.79	0.89	0.39	0.15	9.79	2.72	10.58	3.56	−1.03	0.32	0.25
Sway Area(mm^2^)		295.64	259.08	402.19	614.26	−1.05	0.31	0.23	295.86	274.61	416.13	432.97	−1.33	0.20	0.33

AP: anterior-posterior; ML: medio-lateral; RD: Resultant distance; SD: standard deviation; ES: effect size.

#### With balance perturbation

**Figure 1:**
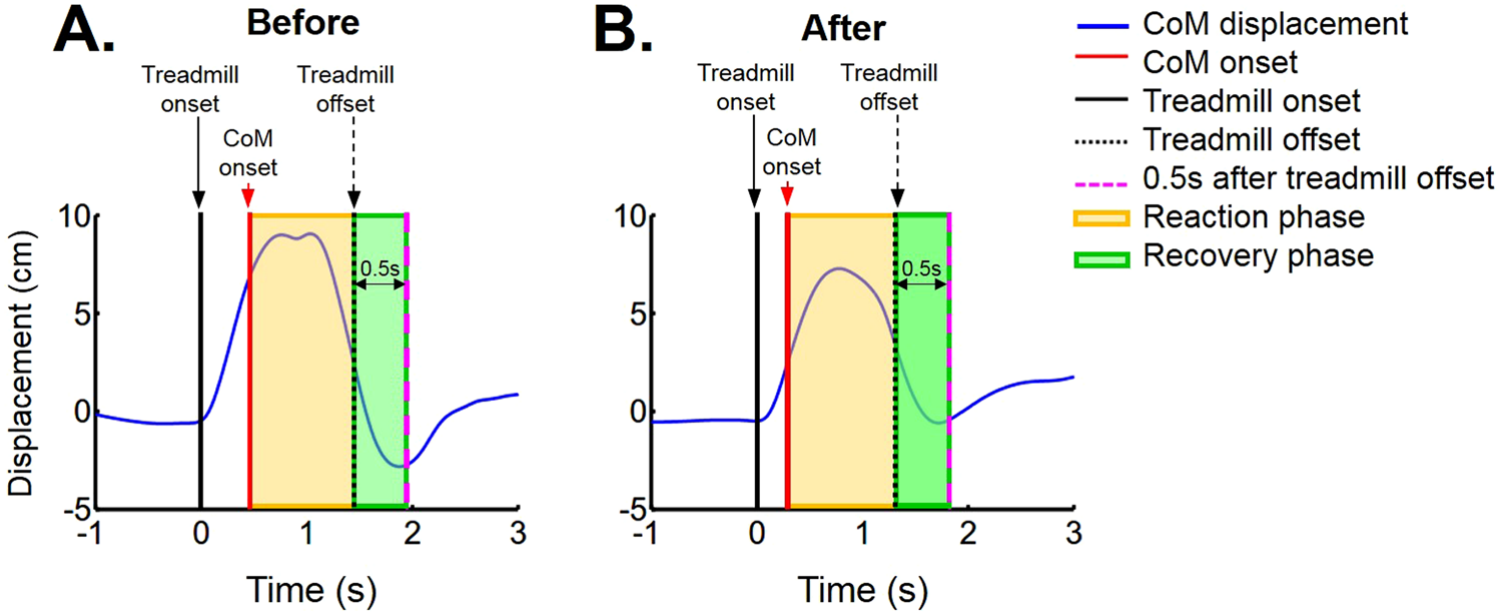
The participant’s center of mass (COM, solid blue line) displacement during the balance response in quiet standing with backward perturbation of a representative participant. (**A**) COM displacement before the training; (**B**) COM displacement after the training. The reaction phase (yellow shaded area) is defined as the time between the COM movement onset (red solid line) and treadmill movement offset (black dot line). The recovery phase (green shaded area) is defined as the time from the treadmill movement offset (black dot line) to 0.5 s after the treadmill movement offset (pink dash line). Note that there were two peaks for COM displacement during the reaction phase before training (**A**), while there was only one peak during the reaction phase after training (**B**). The two peaks of COM displacement might indicate the participant moved around their body to maintain their balance. Therefore, the variance of COM displacement was calculated to quantify both the amplitude and the variability of the COM movement.

However, during quiet standing with perturbation (dynamic condition), the COM variances significantly decreased in the forward, backward, and lateral perturbation trials in the recovery phase after the training (Table 2). A representative participant’s COM data for backward perturbation trial are shown in Fig. 1. The COM displacement in the reaction phase and recovery phase both seems to be decreased after training.

**Table 2:** Variance of center of mass (COM) position during quiet standing with perturbation (dynamic condition).

(unit: mm^2^)	Before	After	Trend	t	*p*	*ES*
**Forward perturbation**						
Reaction phase	223.74 ± 93.40	192.51 ± 72.35	—	1.25	0.23	0.37
Recovery phase	126.18 ± 66.24	89.51 ± 45.83	↓	2.56	0.02	0.64
**Backward perturbation**						
Reaction phase	251.34 ± 180.59	175.30 ± 130.06	—	1.21	0.245	0.48
Recovery phase	212.20 ± 144.88	104.28 ± 63.81	↓	3.08	0.007*	0.96
**Lateral perturbation**						
Reaction phase	217.75 ± 76.78	182.88 ± 21.21	—	1.99	0.63	0.62
Recovery phase	121.18 ± 58.78	81.33 ± 32.96	↓	3.63	0.002*	0.84

*p < 0.008, ES: effect size.

## Discussion

This study evaluated the effectiveness of an 8-week perturbation-based balance training program in community-dwelling older adults. The participants received the training program on a split-belt custom-made treadmill, which could generate forward, backward, and lateral perturbations during walking. The results supported our hypothesis that the balance control of older adults improved after 8 weeks of perturbation-based treadmill balance training.

### Novel Dynamic Balance Training Program

Many previous studies have investigated the balance training program such as Tai Chi, strengthening, aerobic exercise^38–40^ and have yielded various findings. Some of them have shown no improvement in strength or balance control, whereas others have shown improvements in both parameters. Moreover, a meta-analysis of exercise interventions aiming balance improvement suggested that endurance and strength training might not enough to reduce the risk for fall^41^.

The split-belt custom-made treadmill has recently been developed to provide postural perturbations during walking. It can generate unexpected accelerations forward or backward to simulate a slip or a trip and can also generate sideway displacements at various speeds to compromise the lateral stability of participants. The use of external perturbation is thought to train involuntary postural reactions^18,42^, which are key to reducing falling in older persons when balance is disturbed externally in daily life. The use of random multidirectional perturbations during treadmill walking would train the protective reactions of participants in a task-oriented approach^43,44^.

### Enhancement of Static Postural Stability

The COP is widely used to assess static postural stability in people with or without a balance disorder and to quantify their difficulty in performing balance tasks^23–28,45,46^. Previous studies of community-dwelling older adults have reported improvements in COP parameters of static postural stability after conventional balance training, including muscle strengthening, stretching, and balance exercises^25,27,46^. However, in a perturbation-based balance training study using a tilting platform, during quiet standing, COP parameters of static balance in people with Parkinson’s disease did not significantly change, consistent with our results^45^. Hence, during quiet standing without balance perturbation, COP parameters may not be suitable for assessing static postural stability after perturbation-based training. Other non-traditional measures, such as multi-scale sample entropy can extract information associated with a risk of falls^47^.

The COM is a kinematic parameter that can be used to assess postural stability during balance perturbation^6,18,48–50^. The assessment of COM control factors is useful not only for identifying fall risk, but also for specifying target areas for clinical interventions to prevent falls. Previous studies have shown the immediately positive effect of a single slipping (forward) perturbation training session on the postural stability of young adults^48,50^. Moreover, the training effect on postural stability can last for more than 1 day in both young and older adults^6^. Therefore, the improvement of postural stability after forward perturbation training is consistent with our results. Furthermore, our results also showed the training effects on postural stability in the lateral and backward perturbation directions.

### Limitations

The present study has several limitations. First, the broad application of our study findings may be limited due to the lack of an active treatment control group. Second, the older adults enrolled in this study consisted most non-fallers who had high balance performance. Only 2 out of 17 participants in this study had experienced a fall during the last 12 months. Therefore, the training protocol may need to be modified in the future when applying to a population with a high risk of falling by decreasing acceleration and movement speed of treadmill. Additional studies should investigate the training effects in populations with a high risk of falling as well. Moreover, the current study did not compare the training effect of purely medial-lateral perturbation versus purely anterior-posterior perturbation. Future studies can have different groups to do the single perturbation direction training for the group comparison.

### Conclusion and Perspectives

This study evaluated the effectiveness of an 8-week perturbation-based balance training program in community-dwelling older adults. The results showed that after training, the COM control of the older adults was significantly improved during quiet standing with perturbation, while the COP control during quiet standing without perturbation was not changed. The perturbation-based balance training exerted a positive effect on dynamic balance control in older adults. The unique contribution of this study lies in integrating an explicit functional training and biomechanical approach to a relevant clinical problem into a translational model. The findings provides a new paradigm of training guidelines for balance control and can reduce the risk of falling.

## Methods

The study was a single-group intervention with pretest–posttest design and investigated the static and dynamic balance performance of community-dwelling older adults after 8 weeks of perturbation-based treadmill balance training. This research adhered to the principles of the Declaration of Helsinki for human research and was approved by the Research Ethics Committee of National Taiwan University Hospital. The trial was also registered at ClinicalTrials.gov (NCT02821091), and the date of registration was 29/06/2016. Written informed consent was acquired from each participant before they entered the study.

### Participants

Older adults who voluntarily replied to an advertisement posted in a sports center in Taipei were recruited to this study. The inclusion criteria were (1) aged between 60 and 80 years; and (2) ability to stand and walk for 5 min independently without an assistant. The exclusion criteria were (1) neurological disorders or balance difficulties such as vertigo, poor vision, dizziness, stroke, or epilepsy that may prevent standing for the duration of the testing procedures without the aid of an assistive device; (2) pre-existing major lower extremity pathologies such as chronic ankle instability or severe osteoarthritis; and (3) health conditions such as heart disease, uncontrolled hypertension, and chronic obstructive pulmonary diseases that may prevent participation in a balance training program. According to the preliminary result from the 6 pilot participants, the sample size estimation of current study is 15 by calculating the variance of COM during quiet standing perturbation.

### Perturbation-based Treadmill Balance Training

**Figure 2:**
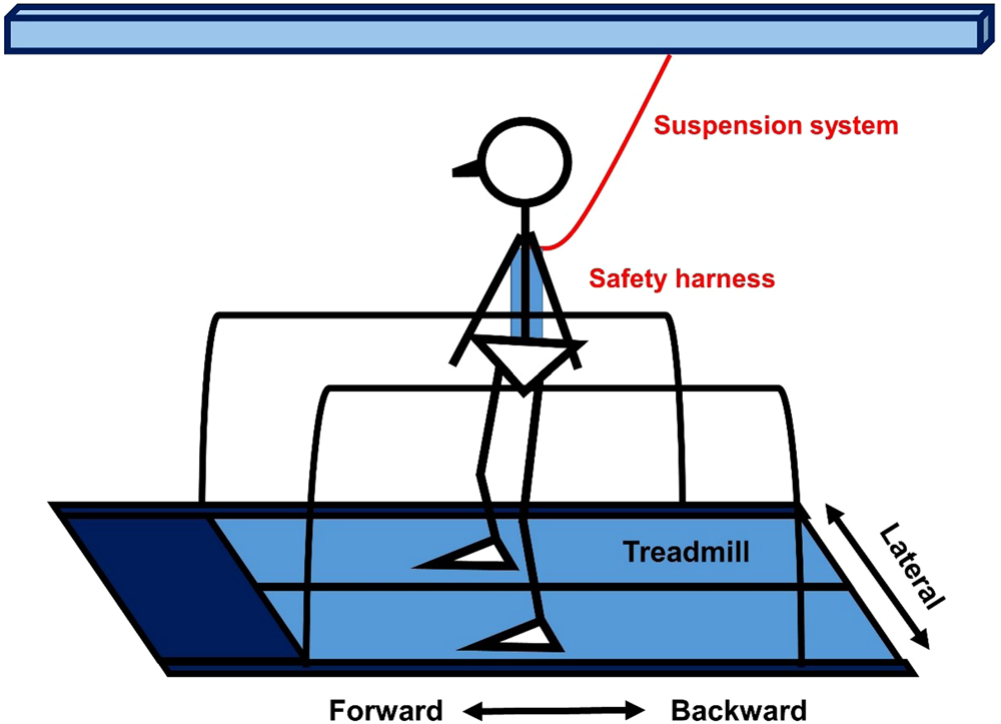
Quiet standing with perturbation.

The participants received 1 hour perturbation-based training that included warm-up, stretching exercises, and walking on a split-belt custom-made treadmill (Fig. 2) for 2 sessions per week over an 8-week period. Since the gait spatiotemporal parameters may be affected by the walking speed^51^, the baseline walking speed was determined based on the walking speed during the 10-Meter Walking Test before training. The participants wore a suspension safety harness; the length of the harness was adjusted to prevent the participants from having any physical contact with the treadmill in case of a fall.

The training protocol continued with the perturbations during quiet standing and walking consisting of 20 forward and 20 backward perturbations, and 40 lateral perturbations (20 times for right to left and 20 times for left to right) in random order for each training session. During quiet standing, the speeds of perturbation were set at 0.15~0.2 m/s in forward perturbation, 0.20~0.25 m/s in backward perturbation, and 0.09~0.18 m/s in lateral perturbation. During walking, the speeds of perturbation were set at 0.5~0.6 m/s in forward perturbation, 0.4~0.6 m/s in backward perturbation, and 0.09~0.18 m/s in lateral perturbation. The detailed settings of the split-belt custom-made treadmill are illustrated in Table 3.

**Table 3:** Individual Split-belt balance perturbation treadmill training protocol.

Tasks	Perturbation (times)	Speed for walking and balance perturbation (m/s)							
		Week 1	Week 2	Week 3	Week 4	Week 5	Week 6	Week 7	Week 8
Walking for 3 min (warm- up)	None	0.8 PWS	0.8 PWS	0.9 PWS	0.9 PWS	1 PWS	1 PWS	1 PWS	1 PWS
Quiet Standing	Forward (20)		+0.15		+0.15		+0.20		+0.20
	Backward (20)		−0.20		−0.20		−0.25		−0.25
	Lateral (40)	0.09		0.135		0.180		0.18	
Walking for 5 min	Treadmill speed	0.8 PWS	0.8 PWS	0.9 PWS	0.9 PWS	1 PWS	1 PWS	1 PWS	1 PWS
	Forward (20)		+0.50		+0.50		+0.60		+0.60
	Backward (20)		−0.40		−0.50		−0.60		−0.60
	Lateral (40)			0.135		0.18		0.18	
5 min rest	None	Rest							
Quiet Standing	Forward (20)	+0.15		+0.15		+0.20		+0.20	
	Backward (20)	−0.20		−0.20		−0.25		−0.25	
	Lateral (40)		0.135		0.135		0.18		0.18
Walking for 5 min	Treadmill speed	0.8 PWS	0.8 PWS	0.9 PWS	0.9 PWS	1 PWS	1 PWS	1 PWS	1 PWS
	Forward (20)			+0.50		+0.50		+0.60	
	Backward (20)			−0.40		−0.50		−0.60	
	Lateral (40)		0.09		0.135		0.18		0.18

Note 1: The number inside the table indicate the speed for walking and perturbation.

Note 2: Preferred walking speed was abbreviated as PWS.

### Data Collection and Instruments

The participants’ characteristics of body height, body weight, and history of falls were recorded. To obtain the fall profile of each participant, fall frequency was established through self-report retrospectively obtained over the past 12 months prior to inclusion in the study. The Mini-Mental State Examination was used to evaluate the cognitive function of the participants^52^.

A three-dimensional motion analysis system with 10 infrared-sensitive cameras (VICON Bonita, Oxford metrics, UK) was used to collect kinematic data. Fourteen-mm spherical reflective markers were attached to the body using a Plug-In-Gait marker set^53^, to create a 15-segmant model for COM kinematic calculation in Vicon Nexus software (ver. 1.8.5, Oxford Metrics Ltd., Oxford, UK)^54^. Kinetic data were collected using three force plates (model OR6-7, AMTI, MA, USA). Kinematic and kinetic data were synchronised, and the sampling rates were set at 120 and 960 Hz, respectively.

### Experiment Protocol

Two quiet standing tasks were performed during the experiments. First task was quiet standing without perturbation (static condition). The older adults stood barefoot with feet-shoulder-width apart on the ground for 60 s with their eyes open and eyes closed. Feet position were marked to ensure identical positioning for each trial. Second task was quiet standing with perturbation (dynamic condition). The participants stood barefoot on a split-belt custom-made treadmill with their feet shoulder-width apart in an upright position for the perturbation trials (Fig. 2). They were instructed to recover from a balance perturbation by returning to their initial upright position, with their feet without moving before and after the perturbation. The speeds of perturbation were set at 0.15 m/s in forward perturbation, 0.2 m/s in backward perturbation, and 0.18 m/s in lateral perturbation to make sure that people had the possibility of using an in-place strategy without stepping. The direction of perturbation during quiet standing tasks were in random order.

### Data Analysis

Vicon Nexus Plug-In-Gait biomechanical modelling program was used to process and output kinematic and kinetic data, including the COM and COP. Kinetic data were calculated using the inverse dynamic approach. A program custom written in MATLAB was used to calculate kinematic and kinetic data. Kinematic and kinetic data were low-pass filtered in MATLAB, with 3 and 6 Hz as cut-off frequencies, respectively.

During quiet standing without perturbation (static condition), the COP excursion, mean velocity, and sway area were calculated using the force and moment recorded by the force plate in the anterior-posterior (AP) and, medio-lateral (ML) directions, and over the resultant distance (RD)^29^. The COP excursion is defined as the total length of the COP path. The COP sway velocity is defined as ratio between the COP travelled distance and the total time of acquisition. The COP sway area is defined as the area of the 95% bivariate confidence circle, which is expected to enclose approximately 95% of the points on the COP path^29^.

During quiet standing with perturbation (dynamic condition), the whole-body COM position combined the AP and ML directions (resultant) was calculated based on the marker position. Because the participants showed some natural sway during quiet standing, the onset and offset events of the COM movement and the balance perturbation (i.e., split-belt motion) were defined as the time of the velocity changes exceeded or fell below the threshold^30^. The threshold was mean ± 3 times of standard deviation of the 1 second of baseline value of COM velocity or split-belt velocity.

The reaction phase was defined as the time between the COM movement onset and the treadmill movement offset, whereas the recovery phase was defined as the time from the treadmill movement offset to 0.5 s after the treadmill movement offset (Fig. 1). Because the balance perturbation was created using a split-belt treadmill, this mechanical translation triggered the movements of the participants passively and generated a passive response component in the participants’ bodies. Thus, the active recover component that was a corrective response came later. The 0.5 second fixed recovery phase was chosen based on the averaged active response time in the previous studies^55,56^. The COM variance during the reactive phase and recovery phase was calculated which can quantify both the amplitude and the variability of the COM movement. The quality of marker data was poor for two participants during the forward perturbation trials; therefore, these data were disregarded.

### Statistical Analyses

The normality of the distribution for the participants’ demographic data was evaluated according to their skewness and kurtosis using the Shapiro-Wilk test. The significance of changes between before and after training was determined using a paired t-test. The level of significance was adjusted by Bonferroni correction (α/6) to avoid Type I error. The significance level was set at 0.008. The sample size was estimated using the power analysis of preliminary data. A significance criterion of 0.008 and power of 0.8 are chosen to estimate the sample size. Effect size as standardized mean difference was estimated by calculating the between-time difference in means at before and after training, dividing by the mean SD at baseline. In interpreting the effect sizes, we used Cohen’s general guidelines for interpretation of strength: 0.2 = small effect, 0.5 = moderate effect and 0.8 = large effect. Statistical analyses were conducted using SPSS 18.0 for Windows.

## Electronic supplementary material

Perturbation Training Video
